# Type 1 Bartter syndrome presenting as primary diabetes insipidus: a rare Case Report with 8-year follow-up

**DOI:** 10.3389/fgene.2025.1672214

**Published:** 2025-11-28

**Authors:** Huijuan Lu, Shaojun Liu, Jia Sun, Lijun Mou

**Affiliations:** 1 Department of Nephrology, Linping Campus of Second Affiliated Hospital, Zhejiang University School of Medicine, Hangzhou, Zhejiang, China; 2 Department of Nephrology, Huashan Hospital, Fudan University, Shanghai, China; 3 Division of Nephrology, The Second Affiliated Hospital, Zhejiang University School of Medicine, Hangzhou, Zhejiang, China

**Keywords:** Bartter syndrome, nephrogenic diabetes insipidus, SLC12A1 gene, focal segmental glomerulosclerosis, renal insufciency

## Abstract

Type 1 Bartter syndrome (BS), a rare autosomal recessive salt-losing tubulopathy, classically presents with hyponatremia, hypochloremia, hypokalemic alkalosis, and hyperreninemic hyperaldosteronism. We report a male patient with the atypical presentation of hypernatremia-hyperchloremia, initially misdiagnosed as nephrogenic diabetes insipidus (NDI). Born preterm with antenatal polyhydramnios, he exhibited persistent hypokalemia, polydipsia, and polyuria despite potassium supplementation. Seizures developed at age 9. Genetic testing revealed compound heterozygous *SLC12A1* mutations, confirming type 1 BS, while excluding primary NDI (both *AVPR2/AQP2* variants were negative). Indomethacin normalized electrolytes and resolved polyuria. Nonadherence to follow-up protocols was observed, including the patient’s unsupervised cessation of indomethacin therapy by the patient at age 14 years. Subsequently, following this unsupervised treatment cessation, he developed focal segmental glomerulosclerosis (FSGS) with renal insufficiency by age 17, alongside intracranial calcifications. This case underscores the fact that hypernatremia-hyperchloremia in BS presenting as secondary inherited nephrogenic diabetes insipidus (siNDI) may mimic primary NDI, and refractory hypokalemia should prompt evaluation for BS. Long-term BS monitoring is critical, given the potential for secondary complications such as FSGS and renal insufficiency, particularly in the context of treatment nonadherence.

## Introduction

Bartter syndrome (BS) is a rare autosomal recessive inherited salt-losing tubulopathy (SLT). BS is classified into five subtypes based on underlying genetic defects, which cause significant variations in clinical manifestations (Konrad et al., 2021). The classical clinical manifestations of both type 1 and type 2 BS include polydipsia, hyponatremia-hypochloremia, hypokalemic metabolic alkalosis, hypercalciuria, and nephrocalcinosis (Konrad et al., 2021; Seyberth and Schlingmann, 2011). In contrast, persistent hypernatremia-hyperchloremia in BS is exceedingly rare. To our knowledge, only six cases with molecular confirmation have been documented in the medical literature to date (Bettinelli et al., 2000; Bockenhauer et al., 2010; Lee et al., 2010; Chuang et al., 2016; Vergine et al., 2018; Ghosh et al., 2016) (Table 2). Nephrogenic diabetes insipidus (NDI) is characterized by the inability of the kidneys to respond to the hormone arginine vasopressin (AVP), leading to the failure of concentrating urine (Kavanagh and Uy, 2019). The development of secondary inherited nephrogenic diabetes insipidus (siNDI) in BS results from a combined failure of the kidney’s concentrating mechanism. The primary defect in BS impairs the creation of a concentrated medullary gradient. Concurrently, the classic BS features of chronic hypokalemia and hypercalciuria directly impair the final step of water reabsorption by downregulating and inhibiting the aquaporin-2 (AQP2) water channels in the collecting duct, rendering it resistant to AVP. Here, we describe a patient with siNDI due to type 1 BS, successfully managed with indomethacin and potassium supplementation. However, the subsequent unsupervised cessation of indomethacin therapy contributed to the development of focal segmental glomerulosclerosis (FSGS) and renal insufficiency, highlighting the critical consequence of suboptimal long-term management.

## Case report

A 1-year-old boy visited the outpatient clinic because of vomiting, polydipsia and polyuria, and he was found to have hypernatremia-hyperchloremia and hypokalemia. The patient was born prematurely due to polyhydramnios beginning in the second trimester, with a birth weight of 1,300 g. His parents were healthy and nonconsanguineous. He was hospitalized at a local hospital, where laboratory investigations confirmed hypokalemia (2.83 mmol/L), hypernatremia (162.6 mmol/L), and hyperchloremia (123.2 mmol/L). Treatment with potassium chloride and spironolactone was initiated. However, after discharge, his symptoms did not significantly improve, polyuria persisted (urine volume: 4–5 L/day), and later in childhood, his growth was observed to be within the lower percentiles but followed a stable trajectory, without evidence of severe growth failure. Pubertal development was noted to have begun in early adolescence and appeared to progress normally. His academic performance was consistently poor. Although a formal neuropsychological evaluation was not conducted, the observed academic struggles likely reflect a combination of factors including chronic fatigue from polyuria/polydipsia, school absenteeism for medical appointments, and the potential neurocognitive effects of recurrent electrolyte disturbances. Seizures developed at age 9 years, prompting his visit to our outpatient clinic.

**FIGURE 1:**
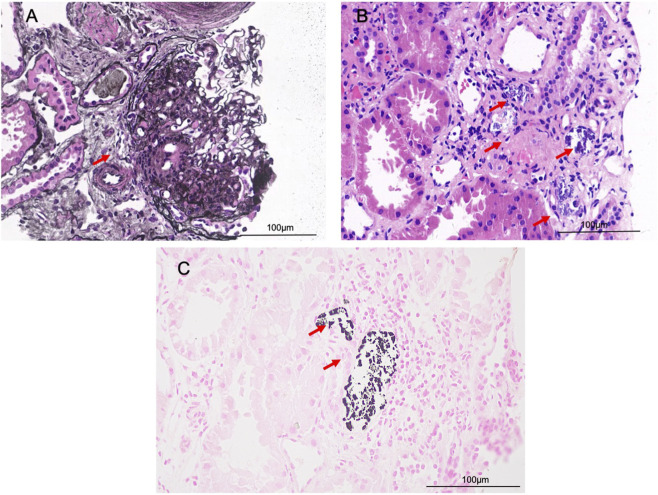
Renal pathology. **(A)** showing focal segmental glomerulosclerosis (red arrow, PASM×400); **(B)** showing calcium phosphate deposition (red arrow, HE×400); **(C)** von Kossa stain demonstrating black calcium deposits within renal tubule (red arrow, von Kossa stain ×400).

**FIGURE 2:**
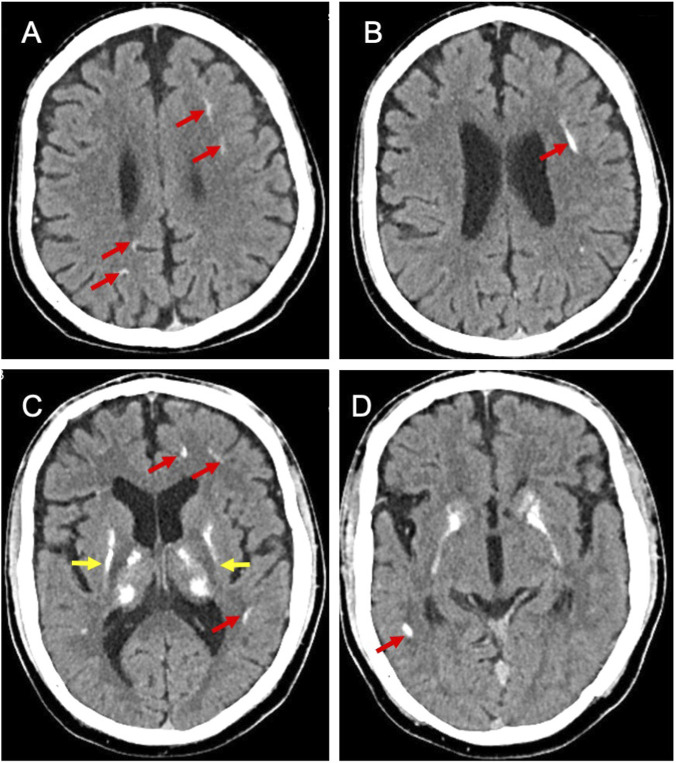
Computed tomography scan of the brain shows intracranial calcifications. **(A)** calcifications in the left frontal subcortical white matter and the right parietal gray-white junction near the cortex (red arrow); **(B)** calcifications in left frontal and parietal subcortical white matter (red arrow); **(C)** calcifications in bilateral basal ganglia regions (yellow arrow), the left frontal and temporal subcortical white matter (red arrow); **(D)** calcifications in the right temporal gray-white junction (red arrow).

**FIGURE 3:**
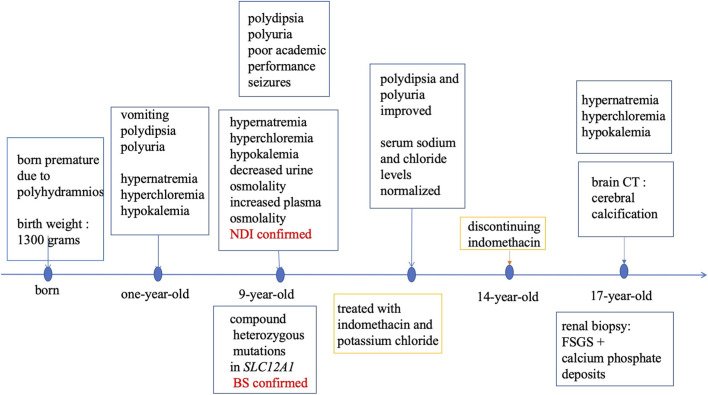
Timeline for diagnosis and treatment.

His growth was normal, his blood pressure was 115/55 mmHg, and laboratory tests revealed hypokalemia due to renal potassium wasting, hypernatremia-hyperchloremia, decreased urine osmolality and increased plasma osmolality (Table 1), findings consistent with diabetes insipidus. The 24-h urinary calcium measurement was significantly elevated at 6.21 mmol/24 h, confirming the diagnosis of hypercalciuria. Plasma renin activity and aldosterone levels were markedly elevated. Plasma renin activity was measured by radioimmunoassay. Plasma aldosterone concentration was measured by chemiluminescent immunoassay. A formal water deprivation test was contraindicated due to safety concerns, but the spontaneous biochemical profile was deemed diagnostic. Renal ultrasonography was normal. Although refractory hypokalemia is not a typical feature of primary diabetes insipidus, the presence of hypercalcinuria and hyperreninemic hyperaldosteronism (Table 1) prompted consideration of BS. Therefore, gene sequencing (targeting 98 genes, including *SLC12A1, KCNJ1, CLCNKB, BSND, CASR, AQP2,* and *AVPR2*) was performed. Gene sequencing revealed compound heterozygous mutations in *SLC12A1*: the patient and his mother had a heterozygote deletion in exons 3-8, while the patient and his father carried a heterozygous frameshift mutation (c.2493-2494del, p. R833Ifs*15). These findings confirmed type 1 BS. No mutations were detected in the *AVPR2* and *AQP2* genes. The combination of genetic findings and the clinical phenotype confirmed a final diagnosis of Type 1 BS with siNDI. Three months after treatment with indomethacin (6.25 mg twice daily) and potassium chloride (3.0 g per day), the patient’s serum sodium and chloride levels gradually normalized, and polydipsia and polyuria significantly improved. However, seizures persisted. The patient demonstrated nonadherence to the follow-up schedule and discontinued indomethacin without medical supervision at age 14 years. He returned for a follow-up visit to our hospital at age 17 years. Laboratory tests revealed hypokalemia and hypernatremia, notably accompanied by the new finding of hypocalcemia (1.98 mmol/L), along with persistent hypercalciuria. The patient also had proteinuria (7685.0 mg/24 h) and a markedly elevated serum creatinine level (214.1 μmol/L) (Table 1). A comparison of laboratory values during successful treatment at age 13 with those after treatment cessation at age 17 illustrates the consequences of nonadherence: well-controlled electrolytes and minimal polyuria gave way to hypernatremia, hypokalemia, and a marked decline in renal function. Renal biopsy was performed, revealing focal segmental glomerulosclerosis (FSGS) and calcium phosphate deposition (Figure 1). In accordance with the standard of care for proteinuria, an angiotensin II receptor blocker (ARB) was initiated as part of a comprehensive nephroprotective strategy to address the significant proteinuria and counteract the deleterious effects of chronic glomerular hyperfiltration. Additionally, a brain CT scan for seizure evaluation showed intracranial calcifications (Figure 2). The diagnostic and treatment are shown in Figure 3.

**TABLE 1:** Comparison of laboratory test results before and after treatment.

Examination item	Age 9 Initial presentation	Age 13 On treatment	Age 17 Treatment discontinuation	Reference range
Serum biochemistry				
Potassium (mmol/L)	2.83	3.34	3.16	3.50∼5.30
Sodium (mmol/L)	162.6	146.0	148.3	137.0∼147.0
Chloride (mmol/L)	123.2	103.1	108.2	99.0∼110.0
Calcium (mmol/L)	2.18	2.18	1.98	2.08∼2.60
Magnesium (mmol/L)	0.87	0.81	0.85	0.70∼1.10
Phosphate (mmol/L)	1.27	1.27	1.82	0.85∼1.51
Albumin (g/L)	44.2	NA	34.9	35.0–52.0
Creatinine (μmol/L)	91.0	86.0	214.1	57.0∼97.0
Upright plasma renin (uIU/mL)	>500	>500	>500	4.4∼46.1
Upright plasma aldosterone (pg/mL)	640	303	1820	30.0∼353.0
Plasma osmolality (mOsm/kg H_2_O)	317	311	309	292–310
Urine osmolality (mOsm/kg H_2_O)	139	164	209	181–1,103
Arterial blood gas analysis				
PH value	7.44	7.486	NA	7.350∼7.450
Base excess (mmol/L)	1.1	6.2	NA	−3.0∼3.0
HCO3− (mmol/L)	24.8	29.7	32.73	22.0∼26.0
PaCO2 (mmHg)	37.1	39.7	NA	36.0∼44.0
Urine analysis				
Urine specific gravity	1.005	1.005	1.005	1.003∼1.030
24 h urine analysis				
Urine volume(L)	4.5	​	5.6	​
Potassium (mmol)	53.1	NA	91.5	25.0–100.0
Sodium (mmol)	81	NA	135.2	130.0–260.0
Chloride (mmol)	94.5	NA	153.9	150.0–250.0
Calcium (mmol)	6.21	NA	9.09	0.25–6.2
Proteinuria (mg/24 h)	157.5	134.4	7685.0	28–141

## Discussion

The pathognomonic features of BS types 1 and 2 comprise polydipsia, hyponatremia-hypochloremia, hypokalemic metabolic alkalosis, hypercalciuria, and nephrocalcinosis. Therefore, hypernatremia-hyperchloremia secondary to BS can lead to misdiagnosis as primary NDI. NDI is characterized by renal unresponsiveness to arginine vasopressin (AVP), which may be hereditary or acquired, resulting in impaired urine concentration (Kavanagh and Uy, 2019). In patients with an early-onset disease and no secondary causes, congenital nephrogenic diabetes insipidus (CNDI)-caused by *AVPR2* or *AQP2* gene mutations-should be suspected. Clinical presentations of NDI include polyuria, nocturia, polydipsia, large amount of diluted urine, and hypernatremia-hyperchloremia. As hypokalemia is a frequent etiological factor in NDI, persistent refractory hypokalemia in these patients warrants comprehensive diagnostic evaluation to elucidate underlying pathophysiological mechanisms.

Secondary NDI associated with inherited diseases can be misdiagnosed as primary NDI. Therefore, the term “secondary inherited nephrogenic diabetes insipidus” (siNDI) has been suggested (Kleta and Bockenhauer, 2018). The concurrent diagnosis of BS with siNDI remains a rare and clinically challenging entity. To our knowledge, only 6 cases have been documented in the medical literature to date (Bettinelli et al., 2000; Bockenhauer et al., 2010; Lee et al., 2010; Chuang et al., 2016; Vergine et al., 2018; Ghosh et al., 2016) (Table 2): five with SLC12A1 mutations (type 1 BS), one with a KCNJ1 mutation (type 2 BS). Common features include hypokalemia (100%), hypercalciuria/nephrocalcinosis (100%), and hypernatremia (50%). This underscores two critical points: First, hypokalemia in NDI necessitates screening for siNDI, particularly BS, especially if hypercalciuria/nephrocalcinosis coexists. Second, hypernatremia is a frequent clue indicating siNDI in BS patients.

**TABLE 2:** Basic information of the 6 patients and the present patient.

Author	Gender	Age	Clinical manifestation								
			Hypokalemia	Hypernatremia	Hyperchloremia	Hypercalcinuria	Nephrocalcinosis	Metabolic alkalosis	Seizures	FSGS	Gene
Bettinelli et al. (2000)	M	20 months	+	+	+	+	+	+	-	-	SLC12A1
Bockenhauer et al. (2010)	F	4 years	+	NA	-	+	+	+	-	-	KCNJ1
Lee et al. (2010)	M	21 months	+	-	-	+	+	NA	-	-	SLC12A1
Chuang et al. (2016)	M	5 years	+	-	-	+	+	+	-	-	SLC12A1
Vergine et al. (2018)	M	1 year	+	+	NA	+	+	-	-	-	SLC12A1
Ghosh et al. (2016)	F	31 months	+	+	NA	+	+	+	-	-	SLC12A1
Lu	M	1 year	+	+	+	+	+	+	+	+	SLC12A1

M, male; F, female; NA, not applicable.

The mechanism of siNDI in Type 1 Bartter syndrome arises from a multifactorial disruption of urinary concentration, rooted in the primary loss of Na + -K + -2Cl- (NKCC2) function (Konrad et al., 2021). This defect not only impairs countercurrent multiplication—thereby diminishing medullary hyperosmolality—but also initiates a predictable sequence of electrolyte disturbances. The ensuing salt-wasting and volume depletion activate the renin-angiotensin-aldosterone system (RAAS), leading to hyperaldosteronism. This, combined with increased distal sodium delivery, potently drives potassium secretion in the cortical collecting duct, establishing the profound hypokalemia characteristic of the disorder (Seyberth and Schlingmann, 2011). Concurrently, the NKCC2 defect abolishes the lumen-positive transepithelial voltage, which is critical for paracellular calcium reabsorption in the thick ascending limb, directly resulting in hypercalciuria (Himmerkus et al., 2025). Critically, these two biochemical hallmarks, hypokalemia and hypercalciuria, act synergistically to induce a functional aquaporin-2 (AQP2) deficiency in the collecting duct, which is central to the siNDI phenotype. Hypokalemia directly reduces the total abundance of AQP2 water channels by promoting their autophagic-lysosomal degradation (Khositseth et al., 2015). Simultaneously, hypercalciuria activates the calcium-sensing receptor (CaSR) in the collecting duct, which inhibits adenylate cyclase and suppresses cAMP-dependent AQP2 trafficking to the apical membrane (Procino et al., 2004; Bockenhauer and Bichet, 2015). This “dual-hit” mechanism—wherein one factor depletes the AQP2 protein pool and the other prevents its membrane insertion—completely abrogates renal water conservation. This pathophysiological sequence explains the paradoxical transition from the classic BS presentation of hyponatremia-hypochloremia to the severe water-losing state of hypernatremia-hyperchloremia that defines siNDI.

This case uniquely presents previously undocumented seizures in this specific condition. Seizures are common in patients with electrolyte disturbances, and persistent hypernatraemic dehydration can cause permanent brain damage. Intracranial calcifications represent another potential etiology for seizures onset in the patient. The mechanisms by which intracranial calcifications lead to epilepsy may involve direct cellular dysfunction, genetic/metabolic abnormalities, and secondary changes from associated pathological processes (Monfrini et al., 2023). In our patient, seizures appear to be associated with both severe long-term hypernatremia and extensive intracranial calcifications. The pathophysiological link between NDI and intracranial calcification has been increasingly recognized in clinical observations (Al-Kandari et al., 2008). Bindu et al. reported a pediatric NDI case with intracranial calcifications and white matter alterations presenting mild spastic paraparesis (Bindu and Kovoor, 2007). Intracranial calcifications in NDI may result from repeated hyperosmolar dehydration causing endothelial injury, and hypernatremia can independently contributes to pediatric neurologic injury and brain necrosis (Bindu and Kovoor, 2007). The seizures and intracranial calcifications observed in our patient, coupled with his documented academic struggles, signal significant central nervous system involvement. This case compels us to look beyond renal parameters and strongly advocate for a new standard of care in the long-term follow-up of BS patients. We propose that comprehensive monitoring must include detailed neurological imaging, preferably with MRI, to identify structural abnormalities such as calcifications, and formal quantitative neurocognitive assessment to objectively quantify any developmental delay or cognitive impairment.

The relationship between BS and FSGS is complex and multifactorial. FSGS development has been reported in some patients with BS (Lee et al., 2011). The authors proposed that the chronic renin-angiotensin system (RAS) stimulation may lead to secondary glomerular hyperfiltration. Other risk factors for renal damage in BS include recurrent dehydration episodes, long-term nonsteroidal anti-inflammatory drugs (NSAIDs) use, and nephrocalcinosis.

The hypocalcemia (1.98 mmol/L) observed at age 17 marks a pivotal shift in calcium homeostasis, signaling advanced Chronic Kidney Disease-Mineral and Bone Disorder (CKD-MBD) (Schmitt and Mehls, 2011). While early-stage BS is characterized by hypercalciuria and nephrocalcinosis, the progression to FSGS and renal insufficiency dramatically altered this balance. The decline in glomerular filtration rate (GFR) led to hyperphosphatemia, which complexes with calcium, and impaired renal synthesis of active vitamin D, which reduces gut calcium absorption (Wesseling-Perry and Salusky, 2013). These factors, triggering secondary hyperparathyroidism, collectively explain the late-onset hypocalcemia and underscore the severity of his end-stage renal disease.

The management of BS and primary NDI presents a therapeutic conflict. NSAIDs such as indomethacin, improve BS symptoms though prostaglandin inhibition but may exacerbate polyuria in primary NDI. Conversely, thiazides used for NDI can worsen BS-associated hypovolemia. We chose indomethacin monotherapy because we hypothesized that the siNDI in BS is mechanistically distinct from primary NDI. Rather than a fixed genetic defect in water conservation, it is a dynamic, reversible phenotype driven by prostaglandin-mediated hypokalemia and hypercalciuria, which secondarily impair AQP2 function. However, subsequent treatment nonadherence precipitated renal function decline, highlighting the critical importance of long-term monitoring and consistent therapy adherence. Our case demonstrates that secondary FSGS from chronic hyperfiltration requires a management paradigm shift in BS. In line with this, we initiated an ARB at age 17 upon detecting significant proteinuria. We therefore advocate for the proactive use of RAS blockade to reduce proteinuria, mitigate hyperfiltration, and provide ancillary hypokalemia management, which is essential for long-term renal protection.

This case report has several limitations. Cognitive impairment was not formally assessed using quantitative measures (e.g., intelligence quotient testing). Furthermore, post-seizure neuroimaging (e.g., MRI) and electroencephalogram (EEG) were not obtained due to financial and social constraints. Critically, the absence of dedicated neurological MRI and formal neurocognitive assessment precludes a definitive understanding of the impact of chronic electrolyte imbalances on brain development and structure. Furthermore, without baseline quantitative neuropsychological data, we cannot objectively quantify the extent of cognitive impairment or its progression, thereby limiting our ability to fully characterize the long-term neurodevelopmental outcomes in this patient. Future studies must include these comprehensive evaluations to fully elucidate the long-term neurological sequelae in such patients.

In conclusion, type 1 BS presenting with siNDI poses a significant diagnostic challenge, primarily due to its atypical hypernatremia-hyperchloremia. Cardinal features promoting evaluation include persistent refractory hypokalemia, hypercalciuria, and nephrocalcinosis. Therefore, clinicians managing NDI with hypokalemia should actively investigate siNDI. Additionally, long-term BS monitoring is critical, given the potential for secondary complications such as FSGS and renal insufficiency, particularly in the context of treatment nonadherence.

## Data Availability

The data that support the findings of this study are available from the corresponding author on reasonable request.
